# SHOULD WE ALLOW ORGAN DONATION EUTHANASIA? ALTERNATIVES FOR MAXIMIZING THE NUMBER AND QUALITY OF ORGANS FOR TRANSPLANTATION

**DOI:** 10.1111/j.1467-8519.2010.01811.x

**Published:** 2012-01

**Authors:** Dominic Wilkinson, Julian Savulescu

**Keywords:** organ transplantation ethics, medical ethics, euthanasia, tissue and organ procurement/ethics, tissue donors/supply & distribution

## Abstract

There are not enough solid organs available to meet the needs of patients with organ failure. Thousands of patients every year die on the waiting lists for transplantation. Yet there is one currently available, underutilized, potential source of organs. Many patients die in intensive care following withdrawal of life-sustaining treatment whose organs could be used to save the lives of others. At present the majority of these organs go to waste.

In this paper we consider and evaluate a range of ways to improve the number and quality of organs available from this group of patients. Changes to consent arrangements (for example conscription of organs after death) or changes to organ donation practice could dramatically increase the numbers of organs available, though they would conflict with currently accepted norms governing transplantation.

We argue that one alternative, Organ Donation Euthanasia, would be a rational improvement over current practice regarding withdrawal of life support. It would give individuals the greatest chance of being able to help others with their organs after death. It would increase patient autonomy. It would reduce the chance of suffering during the dying process. We argue that patients should be given the choice of whether and how they would like to donate their organs in the event of withdrawal of life support in intensive care.

Continuing current transplantation practice comes at the cost of death and prolonged organ failure. We should seriously consider all of the alternatives.

## A. INTRODUCTION

Organ transplantation saves a large number of lives and improves the quality of life of many more. But there is a major shortfall in the availability of organs. This leads to potentially preventable death and morbidity in a large number of people. Yet the resources needed to meet the demand for organs are potentially available. Every day there are a large number of patients, who die in controlled circumstances in hospital, whose organs could potentially save the lives of others. But the vast majority of these organs are buried or burned. (See Box 1: The Case of Ruben Navarro)

**Box 1: The Case of Ruben Navarro^1^**

In late 2006, in a Californian intensive care unit, a 25-year-old man named Ruben Navarro was removed from life support. He had a progressive neurodegenerative disorder, and had suffered an out-of-hospital cardiac and respiratory arrest. He was believed to have sustained significant hypoxic brain injury, and the doctors planned to remove him from the breathing machine and allow him to die. His mother had agreed to Ruben donating his organs after his death.

A transplant surgeon was present when Ruben was removed from life support and directed that he be given doses of sedatives and painkillers. But Mr Navarro did not die quickly enough to meet the criteria for organ donation. In fact he died some eight hours after withdrawal of life support machines. The surgeon involved in the case was subsequently charged with the abuse of a dependent adult (though he was later acquitted). Ruben was not able to donate any of his organs.

The importance of this problem has led in the past to changes in attitude towards, and the legal status and clinical care of dying and dead patients. It contributed to the development and widespread acceptance of brain death criteria. It has led in recent times to the revival of donation after cardiac death. It is the main motivation for proposed changes to consent arrangements for organ transplantation in countries such as the United Kingdom, Australia and New Zealand.

However, if we are to maximize the number and quality of organs for transplantation there are a number of other options that should be considered. We discuss a range of alternatives for increasing the supply of organs from critically ill patients in intensive care from whom it has been decided that life-support should be withdrawn. These options range from changes to consent processes, to the use of pre-mortem extracorporeal membrane oxygenation, or organ donation euthanasia. They conflict with ethical norms governing transplantation to varying degrees. The cost of preserving those norms will be the death or ongoing morbidity of many individuals. This may prompt us to consider whether those principles should be revised or rejected.

## B. THE PROBLEM

In the last 50 years, solid organ transplantation has extended and improved the quality of life of hundreds of thousands of patients with organ failure. 3000 transplants take place every year in the UK,^2^ while there are close to 30,000 solid organ transplants per year in the United States.^3^ Refinements in surgical procedures, immunosuppression and post-operative care for these patients have increased the benefits that organ recipients gain from transplants.^4^ However, many more patients could benefit from organ transplants. There is a critical shortfall in the supply of organs.^5^ There are more than 100,000 patients on the waiting list for a deceased donor organ in the US: in 2007, 18 patients per day died on waiting lists for transplants.^6^ In the UK, 450 patients per year die because of a lack of available organs.^7^ It is likely that this number substantially underestimates the problem since a large number of patients are arbitrarily excluded from waiting lists.^8^ Even larger numbers of patients remain on long-term dialysis for want of a suitable kidney for transplantation. The demand for organs is also rising,^9^ because of the increasing burden of certain types of organ failure (for example due to diabetes), and widening eligibility criteria for transplants.^10^

Recognition of the inadequate supply of organs has prompted a number of countries to contemplate legislative changes that may impact upon organ donation. For example, a number of countries have moved to, or are considering proposals for, opt-out consent systems for organ donation.^11^ It is estimated that such a change would increase the donation rate by 25–30% in the US or UK.^12^ The same considerations have also given rise to changes in the source of organs. The potential for organ donation contributed to the development of brain-death criteria in the late 1960s,^13^ which was central to the development of solid organ transplantation. But brain death is relatively uncommon, and the incidence of brain death is falling. Even if organ donation took place from all patients fulfilling brain death criteria, there would still be insufficient organs to meet demand.^14^

Other sources of organs have been contemplated, including xeno-transplantation, stem-cell-derived neo-organs, living unrelated donors (motivated altruistically, or through an organ market), and individuals in persistent vegetative state or anencephalic infants (See Box 2). These options have been discussed elsewhere.^15^ Some of them may provide sufficient organs to meet the demand in the future, but at present this is not the case.

**Box 2: Sources of organs for transplantation**

- Non-human sources
  - Xenotransplantation
- Human sources
  - Neo-organs
  - Non-vital organs (eg single kidney, part of liver)
    - Living related donors
    - Living unrelated donors
  - Essential organs
    - Permanently unconscious patients (PVS, anencephaly)
    - Life support withdrawal donors (see below)
      - Brain Dead
      - Non-brain dead

Yet there is another significant potential source of organs. A large number of patients die in controlled circumstances in hospitals. In the UK 15000 patients per year die in intensive care,^16^ of whom approximately one third (5000 patients) die following decisions to withdraw or withhold life-sustaining treatment (LST).^17^ It is widely accepted that in the face of extremely poor prognosis it is permissible to withdraw life support and allow such patients to die. Many of these patients could donate organs for transplantation. We will refer to this group as Life Support Withdrawal Donors (LSW donors).

Life Support Withdrawal Donors (LSW Donors):

Patients who are critically ill in intensive care with poor prognosis from whom it has been decided after discussion between doctors and family to withdraw life-sustaining treatment and allow them to die.

We intend this group to include both patients who currently meet the criteria for brain death and those who do not. For the purposes of this paper we set aside the question of whether a whole brain, or higher cortical definition of brain death should be used.

A similar (though much larger) group of Life Support Withholding Donors could be identified – those who will die because life support will not be provided. The same principles could be applied to them, though in practice the probability of survival when life support is withheld may be higher than that for patients in intensive care having life support withdrawn.^18^ In this paper we willfocus on Life Support Withdrawal Donors. This group of patients are presently considered eligible to donate their organs and actions leading to their deaths are widely thought to be acceptable. We do not claim that the alternatives discussed below are the only way to address the problem of the organ shortfall. We set aside the question of whether other sources of organs (Box 2) could provide equivalent or greater numbers of organs.

A typical setting for a LSW donor would be a patient like Ruben Navarro who has suffered an out-of-hospital cardiac arrest, who has been resuscitated and stabilized, but who has suffered severe hypoxic brain injury. The patient does not meet criteria for brain death, but remains ventilator-dependent, and on the basis of clinical and electrophysiological criteria is felt to be certain to die or to survive in a vegetative state.^19^

It is permissible to withdraw life support from LSW donors. Their death is inevitable. Yet the decisions we make about them determine whether their organs will be buried or burned with them, or whether they will be used to save the lives of others. The organs from this group of patients have the potential to alleviate or eliminate the shortfall in organ supply. We will first consider current practice for organ donation from LSW donors.

## C. CURRENT PRACTICE FOR ORGAN DONATION AFTER WITHDRAWAL OF LIFE SUPPORT

Currently organ donation is possible from two subgroups of LSW donors. Patients who are diagnosed as being brain dead may have their organs removed while residual body functions are maintained mechanically. Other patients who are not brain-dead have life support withdrawn. After their hearts stop, such patients are certified as dead and their organs can be retrieved (this is referred to as Donation after Cardiac Death, DCD).^20^ DCD has been estimated to increase the total number of organ donors by upwards of 30%.^21^

Yet DCD is associated with a number of practical limitations. While donation after brain death allows transplantation of both abdominal and thoracic organs, DCD usually yields only the former.^22^ Solid organs rapidly develop ischemic injury if their blood and oxygen supply is compromised. So if patients have a prolonged period of low blood pressure or low oxygen levels prior to death, or if retrieval of organs is not possible expeditiously *after* death, there is a significant decline in the suitability of organs for transplantation. Most guidelines indicate a maximum period between withdrawal of life support and death, after which patients become ineligible for DCD. For example, some recommend that the liver only be retrieved if death ensues within 30 minutes after withdrawal of LST, while the kidney and pancreas may be still retrieved if death occurs within 60 minutes.^23^ Organs must also be removed very soon after death. Donors often have LST withdrawn in the operating suite, with transplant surgeons at hand. This can cause disquiet or distress to family members who wish to be with the patient after LST is withdrawn. It also means that family cannot remain with the patient after death.^24^ Some have suggested that there may be a trade-off between optimal end-of-life care and organ donation.^25^

There have been a number of developments in DCD that improve the viability and number of organs for transplantation. Interventions, both prior to and following death, increase the chance of organs being suitable. Infusion of drugs such as heparin prior to death delays the development of blood clots in organs after circulation ceases. Arterial and venous catheters can be inserted while the circulation is still intact, allowing infusion of cold fluid after death, or making it possible to start heart bypass machines immediately after death is declared.^26^ Reduction in the time-limit for declaring cardiac death improves the chance that certain organs can be used. For example, in recently reported cardiac transplants from DCD donors, surgeons waited only 1.25 minutes after the heart had stopped before commencing organ retrieval.^27^ Following death, donors can be put onto cardio-pulmonary bypass (extracorporeal membrane oxygenation, ECMO), which restores circulation to organs and allows unhurried organ retrieval.^28^ Alternatively organs can be rapidly removed and put onto bypass machines outside the donor's body (ex-vivo ECMO).

Some of these developments, however, have also caused ethical concern. Pre-mortem interventions have been criticized, since they offer no benefit to the patient (the donor).^29^ It has been suggested that they may harm the donor, for example, by subjecting them to painful procedures with insufficient analgesia or leading to complications such as limb ischaemia or bleeding, or that they could potentially hasten death.^30^ And post-mortem procedures that restore the circulation, or transplantation of the heart, raise questions about the definition of death. For example, if circulation is able to be artificially restored after death it cannot be said to have irreversibly ceased and the patient is not by definition dead.^31^ In addition, brain death normally follows rapidly after the circulation has stopped. So patients who fulfill cardiac criteria for death usually fulfill brain criteria soon afterwards.^32^ But if post-mortem ECMO reinstates circulatory integrity it may prevent the development of brain death, creating the unusual situation of a patient who is ‘cardiac dead’ but not brain dead.^33^ It may also lead to the heart re-starting as it is reperfused with oxygenated blood.^34^ To address these concerns some surgical teams have either given boluses of lignocaine (thereby preventing the heart from beating) or devised forms of cardiopulmonary bypass that only restore circulation to abdominal organs.^35^

These unconventional and investigational procedures test the boundaries of the determination of death and the permissibility of organ donation.^36^ They highlight the tension between maximizing the number and quality of organs for transplantation and the care of patients who are dying or dead. But if we are to address the shortfall in organ supply it may be that even more radical changes in organ transplantation would be necessary. We have to decide whether it is worthwhile upholding the principles that currently govern organ transplantation, or whether the unmet needs of patients with organ failure warrant their revision or rejection.

We should first ask what *are* the ethical norms relevant to organ transplantation? Then we will consider alternative strategies for organ donation from LSW donors and the ways in which they may conflict with these ethical principles.

## D. ETHICAL PRINCIPLES GOVERNING ORGAN TRANSPLANTATION

There is a range of ethical principles that are used to determine the acceptability of a proposed organ transplant procedure. (Box 3) There is some overlap between these, and they may come into conflict. We do not have space to provide a normative justification for these principles, nor to specify the relative importance of different principles. It is not an exhaustive list, and there may be others. We merely list here the principles that are most commonly cited.^37^

**Box 3: Ethical principles that are used to determine the acceptability of organ transplantation alternatives**

1. Principle of maximal utility
2. Non maleficence
3. Patient autonomy
4. Family autonomy
5. Dead donor rule
6. Brain-dead donor rule
7. Non-killing

### 1. We should maximize the availability and viability of organs for transplantation (Principle of maximal utility)

As discussed in section B, there are a large number of potential beneficiaries of organ transplants. Solid organs from one donor can be used for up to nine recipients.^38^ The policies for organ transplantation that we adopt affect how many patients will benefit. Other things being equal we should benefit as many of these individuals as possible. We can do this by maximizing the number of donor organs. We should also aim to ensure that those organs are of the highest quality. If the organs are damaged, they are more likely to fail after surgery, leading to the death of the recipient or the need for repeat transplantation. However, there is a need to balance the principle of maximal utility against other considerations including potential harm to donors.

### 2. We should not do things to potential organ donors that may harm them (Non-maleficence)^39^

The generally accepted principle of non-maleficence prohibits doctors from deliberately harming their patients. Certain types of harms are permissible, for example minor harms with the consent of the patient, or harms that are necessary in order to secure a greater benefit. Since the benefit of organ donation is for other individuals, it would not usually be acceptable to inflict a significant harm on the patient in order to facilitate organ retrieval. Minor harms may be acceptable, if the donor has consented to them. Where there are alternative courses of action that involve less harm to organ donors, other things being equal, those should be taken in preference.

If killing the patient were felt to be a harm, then this principle would also justify and overlap with Principle 7 (see below). However, since we are discussing patients for whom it is permissible to withdraw life-support, we assume that death is not necessarily a harm for them.

### 3. We should respect the autonomous wishes of patients regarding organ donation (Patient autonomy)^40^

The autonomy of individuals is valued extremely highly. We generally think it important to respect the wishes of patients who wish to donate their organs, as well as the wishes of those who do not wish to donate. Individuals may have made their wishes explicit before becoming ill – for example by signing up to organ donor registries. Some transplant policies may prevent patients from donating their organs who would like to (or would have wanted to) donate. Other policies may lead to organs being removed when the patient would not have wanted this to occur. Both represent breaches of autonomy.^41^

A related concern is the Kantian rule that prohibits using people merely as a means to an end.^42^ All organ donation could be susceptible to the criticism that it uses people as a means. But where people wish for their organs to be donated, transplantation respects their autonomous will, and hence does not use them as a *mere* means. Indeed, by respecting their autonomous wishes it treats them as an end in themselves.

### 4. We should respect the wishes of the families of organ donors (Family autonomy)

Ultimately most decisions to donate organs are taken at a time when patients themselves are not able to express their wishes. If the patient is not competent, and they have not made relevant advance directives, the patient's family makes decisions about organ donation, reflecting both their beliefs about what the patient *would have wanted* (Principle 3), as well as the family's own preferences for the terminal care of their loved one. The wishes of the family may conflict with the wishes of the patient, for example when a family objects to organ donation despite the patient carrying an organ donor card. The family's wishes are usually respected in such cases, though it is far from clear that this is either ethically or legally justified.

### 5. We should not remove organs from patients prior to death (Dead donor rule)

As defined by some, the dead donor rule explicitly refers to a prohibition on the killing of patients to obtain their organs.^43^ Here we separate out the two components of this norm into Principles 5 and 7. With the exception of donation of non-essential organs from a living patient, it is not thought to be permissible to remove organs from a patient prior to their being declared dead. This may be justified in terms of harms to the patient (Principle 2) – the procedure has no benefit to the patient himself or herself, so it may breach the principle of non-maleficence. It may also be justified in terms of the injunction not to kill patients (Principle 7), since the removal of essential organs is likely to lead to death. Nevertheless there are some conceivable situations where removal of a vital organ would neither harm the patient, nor would it hasten death (see option 5 below). So we have retained this as a separate principle here.

### 6. We should not remove organs from patients prior to brain death (Brain-dead donor rule)

Organ donation usually occurs after brain death. As discussed in the previous section, there are concerns about the possibility of removing organs from patients who are ‘cardiac dead’ but not brain dead. This might be because it is actually brain death that provides the crucial normative justification for Principle 5.^44^ It could also arise from a concern that removal of organs prior to brain death may cause the patient to suffer (Principle 2).

### 7. We should not deliberately kill patients (Non-killing)

There is a strong deontological proscription against the killing of patients. Although there are a few jurisdictions where euthanasia is permitted, most societies hold that doctors should not kill their patients. On the other hand it is almost universally accepted that doctors may withdraw or withhold LST in patients who are dying or who have a very poor prognosis. This is not usually classified as an act of killing on the basis of the doctrine of double effect and the difference between acts and omissions.^45^

## E. POTENTIAL WAYS TO IMPROVE ORGAN SUPPLY USING LSW DONORS

What alternatives are there to existing policies and procedures for organ donation from LSW donors? How would they affect organ quality and supply, and how would they cohere or conflict with the Fundamental Principles of transplantation?

There are two main possibilities. Changes to the processes for obtaining consent would increase the number of organ donors without changing any of the procedures for obtaining organs. Alternatively there a number of possible changes to organ retrieval that would affect the number and viability of organs retrieved. (Box 4, Table 1) Combinations of these are conceivable. As an illustration of the possible effects of these alternatives, we will draw on data from the UK Potential Donor Audit (Table 2).^46^ The figures in Table 2 are approximate, and rely on a number of assumptions that may be challenged. Nevertheless they may be useful to put into context the possible benefits of different policies.

**Table 1:** Organ Donation Options and Principles governing organ transplantation

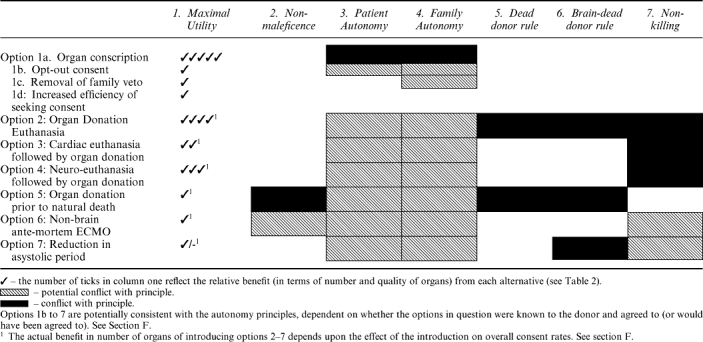

**Table 2:** Estimated potential changes to organ supply in the UK with different options, per year

Options	Number of extra heart-beating donors	Number of extra DCD donors	Total number of extra organs	Proportion of current unmet need
1a. Organ conscription – heart beating only^1^	503	0	1962	4.4
1a. organ conscription – both^1^	503	463	3212	7.1
1b. Opt-out consent	148	33	666	1.5
1c. Removal of family veto^2^	13	6	67	0.1
1d: Increased efficiency of seeking consent	78	218	893	2.0
Option 2: Organ Donation Euthanasia^3^	224–655	−131	520–2201	1.2–4.9
Option 3: Cardiac euthanasia followed by organ donation^4^		93–524	251–1415	0.6–3.1
Option 4: Neuro-euthanasia followed by organ donation^5^	224–655	−131	520–2201	1.2–4.9
Option 5: Organ donation prior to natural death^6^		93–524	158–891	0.4–2.0
Option 6: Non-brain ECMO prior to death		93–524	251–1415	0.6–3.1
Option 7: Reduction in asystolic period^7^	0	0	189	0.4

(See Appendix for an explanation of how these figures were derived. Footnotes in the Table refer to the Appendix.)

**Box 4: Options for increasing the number and quality of organs from LSW donors**

Option 1 – Changes to organ consent processes

Option 2 – *Organ Donation Euthanasia*: Removal of organs from patient under general anaesthesia. Death would follow removal of heart.

Option 3 – *Cardiac euthanasia followed by organ donation*: Euthanasia by administration of anaesthetic and cardioplegic agents. Removal of organs after cessation of circulation.

Option 4 – *Neuro-euthanasia followed by organ donation*: Euthanasia by occlusion of blood vessels to the brain. Removal of organs after brain death certified.

Option 5 – *Organ donation prior to natural death*: Removal of non-vital organs prior to withdrawal of LST.

Option 6 – *Non-brain ante-mortem ECMO*: Cardio-pulmonary bypass to support organs other than the brain and heart prior to withdrawal of LST.

Option 7 – Reduction in asystolic period prior to certification of cardiac death.

### Option 1. Changes to consent processes

Currently in the UK 50% of potential heart-beating (HB ie brain dead) donors, and 13% of potential DCD donors actually donate their organs.^47^ Changes to consent processes could improve this proportion (Table 2). The most dramatic way to increase the conversion rate (the proportion of potential donors who end up donating) would be a form of organ conscription.^48^ Spital and Erin have argued that organ conscription would have distinct advantages in terms of efficiency, cost, and distributive justice.^49^ This would be more problematic in countries without universal health care like the US, where there is already evidence of unfair distribution of organs according to insurance status.^50^ Organ conscription might be applied to individuals who are brain dead, or could be applied also to patients who have died following withdrawal of LST. Thus it has the potential to increase the number of organs for transplantation in the UK by up to 3212 per year. It would violate the principles of Patient and Family Autonomy (Principles 3 and 4), and could be criticized for using patients as a mere means.^51^

Smaller increments in organ donation could be achieved in the UK by moving to an opt-out consent system for organ donation. Some have concerns about whether this would violate the wishes of patients.^52^ However, since this sort of system would allow patients who have a strong preference *not* to donate to opt-out of organ donation, it would appear to respect Patient Autonomy. Most countries that have adopted a form of opt-out consent (including Spain, with the highest donation rates per capita) permit family members to veto organ donation, even where the patient has not opted out during life.^53^ So Family Autonomy would also be respected. A change to an opt-out consent system in the UK would potentially increase the number of transplantable organs by 666 per year.^54^

In a small number of cases families decline organ donation even though the patient had indicated during life that they would like to donate by joining the organ donor register. If families were unable to veto organ donation in such cases this would potentially make available 67 additional organs per year in the UK (Table 2).^55^ This would conflict with the Principle of Family Autonomy.

Alternatively, there may be ways to improve the efficiency of organ donation without changing the nature of consent decisions. Much of the success of the ‘Spanish model’ of organ donation may be attributed to the processes and systems that support organ donation and counsel families.^56^ For example, in the UK potential donor audit there were 141 patients who were diagnosed as brain dead in whom organ donation was not considered, or whose families were never approached.^57^ If all potential HB or DCD donor families were approached for consent (and the consent rates were the same as they are at present), 893 additional organs per year could be made available. This would not violate any of the listed ethical principles governing organ transplantation, but would require significant resources to be made available for counselling and support. This would potentially be sufficient to resolve the current shortfall in organ supply.^58^

### Option 2. Organ Donation Euthanasia

Some have argued that we should reject the ‘dead donor rule’ (Principle 5).^59^ This has mainly been embraced to consider patients who are permanently unconscious, for example those in a permanent vegetative state, or anencephalic infants.^60^ According to some views, their organs can be removed because they have no prospect of regaining consciousness and continued life cannot benefit them.^61^ In James Rachel's terms, while their biological life may continue, their biographical life has ended.^62^ They cannot be harmed by removal of their organs.^63^ However, such a proposal could be more widely cast to include LSW donors.

It is permissible to withdraw life support from a patient with extremely poor prognosis, in the knowledge that this will certainly lead to their death, even if it would be possible to keep them alive for some time. It is permissible to remove their organs after they have died. But why should surgeons have to wait until the patient has died as a result of withdrawal of advanced life support or even simple life prolonging medical treatment? An alternative would be to anaesthetize the patient and remove organs, including the heart and lungs. Brain death would follow removal of the heart (call this *Organ Donation Euthanasia (ODE)*). The process of death would be *less* likely to be associated with suffering for the patient than death following withdrawal of LST (which is not usually accompanied by full anaesthetic doses of drugs). If there were a careful and appropriate process for selection, no patient would die who would not otherwise have died.^64^ Organs would be more likely to be viable, since they would not have sustained a period of reduced circulation prior to retrieval. More organs would be available (for example the heart and lungs, which are currently rarely available in the setting of DCD). Patients and families could be reassured that their organs would be able to help other individuals as long as there were recipients available, and there were no contraindications to transplantation. This is not the case at present with DCD, since many patients do not die sufficiently quickly following withdrawal of LST for organ retrieval.

This proposal has been mooted before.^65^ For example, Robert Truog has argued that those who are imminently dying or permanently unconscious should be permitted to donate their organs in a manner similar to that described above.^66^

ODE would not be likely to cause the patient suffering, however, it would conflict with Principles 5, 6 (the dead donor rules) and 7 (non-killing).

Technically, this would be a form of killing – active euthanasia. It would conflict with the doctrine of double effect. Yet the justification for ODE is wider than that for organ donation from permanently unconscious or brain dead patients. The broader justification for ODE includes not merely those who no longer have interests, but those who will inevitably shortly die. The argument for removing organs from this group is even stronger. It does not rely on controversial judgements of quality of life, wellbeing or interests. These patients will die because they are on life sustaining treatment and it will be withdrawn. Indeed we would suggest that, although most arguments for euthanasia are distinguished from questions of organ donation, it may be that the benefits of donation, for the individual and for others, provide the *strongest case* for euthanasia.

One of the most basic principles of rationality and economics is that if one state of affairs is a *Pareto improvement*, we have strong reason to prefer it.^67^ A change is a Pareto improvement if it leads to at least one individual being better off, while no individual is worse off.^68^ States of affairs in which some individuals are better off while others are worse off are not Pareto improvements. Similarly, changes that involve benefits in some respects, but costs in other respects would not be Pareto superior.

ODE for LSW donors can be regarded as a Pareto improvement to the current practice of withdrawal of life-sustaining treatment and donation after cardiac death. In all cases the patient dies, but in the case of ODE more lives are able to be saved by harvesting functioning organs, and the desire of the patient that their organs be used to help others is more likely to be able to be respected.

ODE might not be regarded as a Pareto improvement if the killing of the patient were regarded as a moral harm or a rights violation.^69^ However, it is difficult to see why a patient is morally harmed or has their rights violated if they are actively killed, compared with a state of affairs where they die as a result of treatment withdrawal, assuming that they have consented to either. Note that while ODE in the case of withdrawal of LSW donors is a Pareto improvement, the same might not be said for removing organs from patients who are permanently unconscious, unless treatment would otherwise be withdrawn. Such patients may survive for some time, and have a finite (albeit small) chance of spontaneous or treatment-induced improvement in consciousness.^70^

It is difficult to estimate the effect of introducing ODE on overall organ donation rates. It could be up to an additional 2201 organs per year in the UK: crucially, however, it would depend upon the consent rates for organ donation and how they are affected by the introduction of this alternative. (See Section F for further discussion of this) Nevertheless it would provide individuals with the greatest possible chance of donating their organs to others. For each patient who would not otherwise be able to donate (because death would be too prolonged following withdrawal of LST), up to nine additional individuals would be able to receive organs.

### Option 3. Cardiac euthanasia prior to organ donation

If we believe that we should not remove organs from patients who are still alive, even where they have consented to this and would otherwise die anyway, then one alternative would be to euthanize the donor and retrieve organs after cardiac death had been declared. This would already be a theoretical option in countries where euthanasia is permitted. Organ donation after cardiac euthanasia has been described in a patient in Belgium.^71^ Organ donors could be given large doses of sedative, and cardioplegic agents (to stop the heart). Again, this would reduce the risk of patients suffering after withdrawal of LST and make organ donation possible for some patients who would otherwise not be able to donate. In an extreme case, they might choose to undergo euthanasia at least partly to ensure that their organs could be donated. However, the agent used to stop the heart, and the period of loss of circulation that ensued, might compromise organ viability in the same way as DCD does at present. The magnitude of the benefit is hard to quantify but it is likely to be less than ODE.^72^

### Option 4. Neuro-euthanasia prior to organ donation

Since patients who donate organs after Cardiac Euthanasia (Option 3) may be able to donate fewer organs (and have more compromised organs) than HB donors, one option would to perform euthanasia in such a way as to induce brain death (we could call this *Neuro-euthanasia*). After sedation and local anaesthesia, catheters could be inserted into blood vessels in the groin of the patient. These could then be advanced to the blood vessels supplying the brain. Catheter occlusion of both internal carotid arteries and vertebral arteries would lead to brain death, while life support would continue to be provided to maintain the circulation to other organs. After brain death was confirmed, organs could be retrieved. There may be concerns about possible discomfort to the patient caused by the neuro-euthanasia procedure; but this could be mitigated by giving the patient a general anaesthetic before the procedure.

But Neuro-euthanasia may affect the viability of organs for transplantation. There are variable criteria for diagnosing brain death across the world.^73^ Many countries require a period of observation to confirm the diagnosis of brain death, which can lead to some delay prior to organ retrieval. Maintenance of circulation and organ viability is not always easy after brain death, and the process of brain death itself may compromise subsequent organ function.^74^ The number and quality of organs potentially available following this procedure would be higher than after Cardiac Euthanasia but less than ODE.

Obviously both Options 3 and 4 would violate the injunction against physician killing (Principle 7) even if they do not violate the dead donor rules (Principles 5 and 6). It is hard to believe, however, that if it is permissible to euthanize a patient who would otherwise die following withdrawal of life support, that Neuro-euthanasia prior to organ donation would be preferable to ODE.

### Option 5. Organ donation prior to natural death

Are there options that do not involve physician killing? It is permissible for living patients to donate non-essential organs. For patients who are dying, or who will die rapidly of cardio-respiratory failure following withdrawal of LST, certain organs are no longer essential. Patients could be anaesthetized and solid organs removed, for example kidney, liver, and pancreas. Removal of these organs would probably not hasten death.^75^ After donation of organs, mechanical ventilation and medications to support the circulation could be withdrawn, and the patient allowed to progress to circulatory death. Other organs may be able to be retrieved after cardiac death was established.

This option would conflict with Principles 5 and 6, the dead donor rules. But since it is permissible to donate non-essential organs (for example in living related or altruistic kidney donation) these rules do not necessarily preclude donation.

If organ donation occurs under anaesthesia it would be unlikely to cause the patient to suffer immediately. However, since patients are sedated but not usually fully anaesthetized at the time when LST is withdrawn, it is possible that after the surgery but prior to death the donor would experience pain that they would not otherwise have experienced.^76^ In addition there are some patients, dependent upon life-support, who survive for a longer period of time after LST is withdrawn. Such patients could experience negative consequences of having organs removed, and their death may be hastened. Pancreas and kidney function could be provided artificially, however, liver function is not able to be readily replaced. This might mean that only kidney and/or pancreas transplants would be permitted prior to natural death.

The numbers of additional organs that would be available from this policy are likely to be significantly less than with Options 2–4. (Table 2)

### Option 6. Non-brain ECMO prior to natural death

Heart-lung bypass (ECMO) has already been used after death to improve the perfusion of organs until they can be retrieved.^77^ It is felt to be acceptable to insert tubes into blood vessels prior to death in DCD donors to reduce delays in initiating bypass.^78^ One option would be to provide a form of organ support prior to death. The bypass machine would support the circulation of all organs except the heart, lungs and brain. Life support (except the ECMO machine) would be withdrawn, and the patient's heart allowed to stop naturally. Once the patient was declared dead, the organs could be removed. The advantage of this procedure would be that organs could be retrieved even if it took some hours for the patient's heart to stop. There would be no unseemly rush after life support was removed, and family could spend time with the patient after death, before organ removal. Non-brain ECMO would not change the status of the patient, since cardiac death would lead to brain death. Non-brain ECMO would not *cause* the patient's death (so it would not necessarily violate Principles 5–7).

It should be noted that although non-brain ECMO would be technically possible, it would be difficult to be sure that it would not alter the progression to cardiac death after withdrawal of LST. ECMO is an expensive and invasive intervention, and could potentially harm the patient (Principle 2). It would potentially lead to similar numbers of organs to Option 3 (Cardiac euthanasia prior to organ donation). Thoracic organs would not be able to be retrieved.

### Option 7. Organ donation following brief asystole

Currently most DCD organ donation procedures commence 5–10 minutes after the heart has stopped (asystole). Some centres commence organ donation earlier, after 2^79^ or even 1.25 minutes of asystole^80^ since this may improve the viability of organs. However, this interval could be shortened further, for example to 20 seconds before declaration of death.^81^

Current definitions of cardiac death are based upon the idea of irreversible cessation of circulation. Although the circulation *could* be restarted with initiation of resuscitation measures after periods of even 10 minutes of asystole, it has been argued that in the context of withdrawal of LST it would not be appropriate (or ethical) to attempt to do so.^82^ A decision has been made to discontinue life support and whatever reasons have justified that decision they would apply equally to not resuscitating. But ‘irreversibility’ is then contingent upon a decision not to attempt resuscitation.^83^ Such a definition of irreversibility might also apply to shorter periods of asystole. But the heart may sometimes start again by itself (so-called *autoresuscitation)* after brief periods of asystole. So this option may hasten death in patients where the heart would have autoresuscitated had not organ retrieval commenced (thus contravening Principle 7).^84^ It would also potentially violate Principle 6 (Brain-dead donor rule) since there would not have been sufficient time after cessation of circulation for brain death to occur.

Option 7 would not lead to any more donors than available at present, though it would increase the viability of organs from DCD donors, and potentially make donation of heart and lungs from such patients possible.

## F. OBJECTIONS TO ORGAN DONATION OPTIONS

The aforementioned alternatives for increasing organ availability may seem disturbing or even shocking. As highlighted, they conflict to some degree with one or more of the fundamental principles governing organ transplantation and the care of dying patients.^85^ Yet many of the currently accepted transplantation practices (donation after brain death, or donation after cardiac death) would have been shocking to the societies of only a few decades ago. The needs of patients who are suffering and dying for want of available organs have previously led us to revise our principles governing transplantation. Perhaps they should again. What specific objections might be raised to them?

### 1. Consent

One objection to most of the above alternative organ donation procedures is that the prior consent of donors may not apply. It is not likely, for example, that current potential donors had imagined that they might be placed on heart bypass machines before their death in order to maintain their organs. A similar criticism has been levelled at DCD as currently practised,^86^ and may have applied in the case of Ruben Navarro, referred to at the start of this paper.

It might not apply to all patients however, and this criticism would not necessarily apply to future donors. If a society adopted Option 2 for example, it may then come to be widely understood that those who consent to organ donation and who are dying in intensive care would have their organs removed in a surgical procedure that would lead to death. The important question for Options 2 to 7 is whether such procedures should be allowed – if consent is given. Furthermore it would be possible to give donors a range of options of organ donation, and the freedom to choose the method that is most consistent with their own values. This would fully respect the autonomy of donors and may increase the acceptability of changes to organ donation practice (see below).

One of the alternatives outlined above is to reject the need for consent for organ donation and to conscript organs (Option 1a). This would require a paradigm shift in approach to organ donation, but as is apparent from Table 2, it is the alternative likely to lead to the greatest increase in the number of available organs. The main reason not to have an organ draft would be that it would violate the autonomy of patients and families who do not wish to donate. We need to weigh this against the substantial burden of death and illness that organ conscription could prevent. At times of great community need (for example during war) conscription of the living for military service has been the norm, and continues to be in some parts of the world. (Organ Conscription would seem a far preferable alternative to military conscription since it would involve no individuals dying or suffering for the sake of others.) But the greatest hurdle to any such change may be community acceptance.

### 2. Community Acceptance?

There is significant public concern about elements of current transplantation practice. So it may be argued that any of the alternatives discussed in this paper (particularly the more radical ones) would be extremely unlikely to meet with general community acceptance (and hence would not be politically achievable). A related concern is that this may threaten acceptance of transplantation more generally.^87^

In the absence of conscription, organ donation is dependent upon the implicit or explicit consent of potential donors. Organ donation options outlined above may alienate or disenchant potential donors. They could *paradoxically* lead to an overall reduction in available organs due to fewer people signing on to organ donor registries, and fewer families consenting for donation.

If it were the case that changes to organ donation procedures led to a fall in consent rates, this would undermine the principal reason that we have given to change donation procedures. However, whether or not this eventuates would depend upon how such a change were instituted, how the consent of patients were sought, and how well public concerns about such a change were able to be allayed. It is not *necessarily* the case that changes to organ donation would lead to a loss of community confidence. For example, one option mentioned in the preceding section would be to allow patients specifically to opt in to the novel procedure or to a range of novel procedures of their choice.^88^ Potential donors could indicate which of several organ donation procedures they consented to (e.g. brain-death donation, DCD, ODE) at the time of signing on to an organ donation registry, or when making advance directives. This sort of specific consent would maximize patient autonomy in relation to organ donation, as well as to helping to allay community fears.^89^

It should be noted though, that this may reduce the effectiveness of the change in terms of increasing the numbers of available organs. It is likely that at least initially the numbers of individuals who would opt-in to ODE (for example) would be small. It would mean that the overall impact of introducing ODE would be much less than the estimates in Table 2. It would undermine the case in terms of organ supply for this alternative. It could, however, be used as proof in principle of the procedure as a way of ethically increasing the supply of organs.

Two other important community fears that would need to be addressed in any change in organ donation policy include patient selection for donation, and harm associated with the procedures.

### 3. Patient selection for organ donation

One concern sometimes expressed about changes to organ donation procedures is that this may lead to the death of patients who would have otherwise survived. If doctors know that a patient's organs can be used to save others' lives, it may influence their assessment of whether a patient's condition is truly hopeless. Of course, this concern might apply equally to current practice of DCD as for Options 5 to 7. It could be thought to apply in the case of Ruben Navarro, for example. It would potentially be a greater concern for Options 2–4, since they would involve actions leading to the patient's death.

Yet it is possible to prevent this from happening. Current practice for diagnosis of brain death, or for decisions about withdrawal of LST, is that these processes are temporally and logistically separated from considerations of eligibility and consent for transplantation. The question of whether or not a patient would have wanted to donate her organs, and whether the family would agree to this, is only raised *after* a decision has already been made to withdraw life support. People not involved in the clinical care of the patient (for example regional transplant coordinators) usually initiate such discussions. And intensive care staff rather than the transplant team provide the care of the patient after withdrawal of LST.^90^ This may not be sufficient to prevent some contamination of decisions about withdrawal of life support – for example if doctors happen to know that a patient is an organ donor. It may be a greater concern if organs are conscripted, since doctors will know with certainty that if brain death is diagnosed, or if a decision is made to withdraw LST, organs will be available to be retrieved.

One additional measure that could prevent abuse and allay concern would be to set up an independent body responsible for confirming brain death or extremely poor prognosis. This group's sole function would be to perform neurological tests and to review prognostic information. They would be called in, as regional transplant teams are currently called in, when a patient was felt by doctors to be a potential organ donor. But they would have no role in transplants, and would be responsible for ensuring that patients with a significant chance of meaningful recovery were not euthanized or allowed to die.^91^

If there were careful selection, including independent scrutiny of decisions, changes to transplantation processes would not necessarily lead to any patients dying who would otherwise have lived. Indeed, such a process may promote more ethical withdrawal of treatment.

### 4. Harm from organ donation

The other fear related to organ donation that would need to be addressed if the community were going to embrace a change in practice, is that of harm or suffering from organ donation. It is one thing for someone to agree to donate their organs after death. At that point, they cannot be harmed by what happens to their body. However, if transplantation practice is changed to include procedures that can take place before death, patients may have some reason to worry about whether they will suffer as a consequence. One fear that people sometimes express about organ donation is of being conscious of having one's organs removed.^92^

If Option 2 (Organ Donation Euthanasia) were adopted, then people might have more reason than at present to harbour this fear. However organ donation would occur under general anaesthesia. There would be no more reason to fear being conscious during this procedure than there is to fear being conscious during any surgical procedure, including the widely accepted practice of live kidney donation. In fact there is some reason to think that options 2–4 would be *less* likely to cause patients to suffer than current procedures for withdrawal of life support and organ donation. On the other hand, Options 5 to 7 could be associated with conscious suffering for the organ donor. This would count strongly against them, or would mandate their modification to avoid this possibility.

### 5. Preferable alternatives

Some may argue that even if it would be, in theory, possible to gain community acceptance of an alternative such as ODE, other alternatives should be pursued first. For example, improved efficiency of obtaining consent, or the adoption of higher brain criteria for brain death may be politically easier to achieve.^93^ As noted at the start of this paper, we do not claim that the alternatives discussed here are the only ways to address the organ shortfall. However, given the ongoing preventable death of large numbers of patients with organ failure, we believe that it is important to consider seriously all the alternatives. Unless, and until these other options alleviate the demand for organs, there is good reason to permit ODE.

### 6. Broader implications

Finally, it may be objected that the arguments advanced above in support of novel organ donation alternatives would support a much broader policy of allowing patients to choose Organ Donation Euthanasia, for example if they were terminally ill or rationally suicidal.

However, although Options 2–4 would conflict with the doctrine of double effect and Principle 7 (Non-killing), it does not follow from the arguments in this paper that other instances of intentional ending of life would be necessarily permissible. ODE, as we have described it, is supported by a basic principle of rationality. It would be a Pareto improvement in that only patients who would have died anyway donate their organs. In contrast, the introduction of a policy of voluntary active euthanasia would not be a Pareto improvement, since it would involve the death of patients who would otherwise have lived.^94^

The arguments developed above may lead some to reject the doctrine of double effect, and consider allowing voluntary active euthanasia. But we have suggested that there are *particularly* strong reasons for allowing individuals to choose how their life ends if they are dependent on life support and life-sustaining treatment is going to be withdrawn. Those who do not wish to support voluntary active euthanasia in other circumstances should consider whether an exception to the doctrine of double effect and the Principle of Non-Killing is justified in this situation.

At the start of this paper we referred to the case of Ruben Navarro. It might be thought that his case highlights the dangers of expanding organ donation processes. Some of the above concerns (including in particular those of consent and patient selection) were expressed in media commentary on the case.^95^ On the contrary, we believe that providing more options to patients, and the reassurance of independent confirmation of prognosis would avoid many of the pitfalls of that case.

## CONCLUSIONS

In this paper we have argued that one of the ethical principles that should influence, and in the past *has* influenced transplantation policy is the need to maximize the number and quality of organs for transplantation. There is a substantial shortfall in organs for transplant. We could overcome this in a range of ways. Future developments in xenotransplantation, stem cell-based therapies, or neo-organs might make the use of organs from deceased donors unnecessary. However, such solutions are some time off, and in the meantime thousands of patients per year die for want of a transplanted organ.

The most promising immediate source of organs for transplantation is the large number of patients who die in intensive care units in hospitals following diagnosis of brain death, or decisions to withdraw LST on the basis of poor prognosis, the group that we have referred to as LSW donors. At present the majority of such organs are buried or burned with the patient.

We have suggested a set of options for increasing the number of organs that could be made available from LSW donors. Simple measures should be adopted, including improved efficiency of approaching families for consent or a switch to an opt-out consent system; however, they may not be enough to resolve the organ shortfall. Organ Conscription would have the greatest potential to increase the numbers of organs available for transplantation, though it would come at the cost of patient and family autonomy. If Organ Conscription is not acceptable, the alternative that would have the greatest potential in terms of organ numbers would be Organ Donation Euthanasia. (Box 5)

**Box 5: Proposal for allowing Organ Donation Euthanasia (ODE)**

**Definition**: Removal of organs from a patient under general anaesthesia. Death follows removal of the heart.

**Eligibility criteria**:

1. The patient is dependent on life support in intensive care
2. Withdrawal of life support is planned on the basis of poor prognosis
3. Death is predicted to occur within a short period after withdrawal of life support
4. Prognosis has been independently confirmed
5. The patient has consented specifically for Organ Donation Euthanasia

**Arguments in favour of Organ Donation Euthanasia**

1. It would promote patient autonomy
2. It would provide patients with the greatest chance of being able to donate their organs after death
3. It would be a Pareto improvement over current practice for treatment withdrawal and increase the number and quality of organs available for transplantation.
4. Suffering or discomfort for the patient would be less likely than with withdrawal of life support

**Arguments against Organ Donation Euthanasia**

1. It may lead to a fall in organ donation rates due to community non-acceptance
2. It could lead to the killing of patients who would not otherwise have died

Organ Donation Euthanasia would conflict with the dead donor rules, and the injunction against physician killing. Yet it would not (if appropriate safeguards were provided) lead to the death of any patients who would otherwise live. The justification for this is not limited to utilitarian considerations. It is a Pareto improvement on current practice for withdrawal of LST and organ donation, and may be Pareto optimal. ODE would apply to patients who are going to die – and soon. It is already accepted that it is permissible to withdraw life support from these patients. It would prevent those individuals from suffering as a consequence of withdrawal of life support. And it would save the lives of up to 9 other individuals. Many potential LSW donors, even if they would have wanted to donate their organs, and their families consent, are currently unable to donate. Their organs die with them.

The most acceptable way to introduce Organ-Donation Euthanasia would be to make it available as an option for prospective organ donors. It must be noted, though, that if ODE were made available in this way, it would have (at least in the short term) only a small impact upon the organ shortfall since only a few individuals would be likely to embrace it. This would undermine to some degree the case in terms of organ supply for ODE. But if we can save even one life, that is something of great moral importance. Many lives could be saved even if only a small percentage of people opted for ODE. And there is also a strong autonomy-based argument for allowing individuals who wish to donate their organs to opt in to ODE (in the circumstance that they are severely ill in intensive care and going to have life support withdrawn.) We should allow people to make advance directives indicating that they would like to be eligible for this alternative. We should encourage and support such altruistic desires.

To some degree at least, there is a conflict between the need to supply organs to the largest number of individuals able to benefit from them, and our beliefs about how we ought to care for those who are dying or dead. We have outlined seven alternatives that may increase the supply of organs. These alternatives clash with one or more of the traditionally accepted ethical principles that govern transplantation, though they potentially promote other ethical values including those of autonomy and beneficence. Whichever transplantation policy is adopted, we should ensure that decisions about withdrawal of life-sustaining treatment are separated from decisions about organ donation, and that organ donation procedures carry minimal risks of causing suffering to organ donors. But continuing current transplantation practice comes at a cost, in terms of a significant number of patients who die or continue to suffer organ failure for want of an available organ. We should think seriously about whether it is time to embrace an alternative strategy.

## APPENDIX

### Estimating the potential benefit of alternatives in terms of organ supply in the UK per year

In 2007/2008 in the UK there were 593 heart beating (HB), i.e. brain dead. donors and 131 DCD donors.^96^ This corresponds to approximately 2312 organs available for transplantation. There are 8000 patients on waiting lists for organs (from a deceased donor), and approximately 450 patients die on waiting lists per year.^97^

We have assumed that HB donors yield 3.9 organs per donor, and DCD donors 2.7 organs per donor.^98^ For Options 2–7 we have assumed that consent rates are similar to current levels in the UK. This assumption may not be valid. See Section F.

Some alternatives could yield more organs than would be needed to prevent the death of patients on waiting lists. Additional organs would reduce the waiting period for transplantation, and may also make it possible for patients previously excluded from transplantation to be offered organs. It is not clear how many patients fall into this latter category, but the number may be considerable.^99^

1. Not all patients who currently consent (or whose families consent) to organ donation end up donating their organs. This might be because their organs are unsuitable for donation (for example if they do not die quickly after withdrawal of LST, or if an unsuspected malignancy is found at the time of organ donation). So the total number of donors with conscription = Total No. Potential donors * conversion rate (for consenting patients)
2. In the UK potential donor audit, 2% of potential HB donors (where the organ donor register was checked) were listed on the register, but their family declined consent for organ donation. 3% of potential DCD donors were registered donors, but their family declined consent. This would potentially translate into a 2.3% increase in HB donors and a 4.5% increase in DCD donors^100^
3. The maximum benefit for organ donation euthanasia depends upon the number of eligible patients. Current patients who are DCD donors would be able to donate more organs (they would become HB donors – this is the reason for the negative net number of DCD donors). There would be additional patients who currently are not eligible to donate by DCD because they do not die soon enough after withdrawal of LST. In the UK Potential Donor Audit 93 potential DCD patients from whom consent was obtained for organ donation did not end up donating. Assuming that these patients fall into this category of slower death, euthanasia prior to organ donation would lead to an additional 93 (HB equivalent) donors. However, this is likely to be an underestimate.^101^ Based upon Swiss data, 80% of potentially eligible patients having withdrawal of LST took more than 1 hour to die.^102^ So an alternative estimate for the number of patients who could be able to donate (following organ donation euthanasia) would be 5 times the current number of donors.
4. This would potentially lead to a similar number of donors as organ donation euthanasia, though the number/viability of organs would be similar to current DCD donors (so the overall number of organs would be less).
5. It is hard to quantify the number of organs that might be available. The maximum number would be close to the number available following organ donation euthanasia, however, the viability of organs may be compromised.
6. This option may improve the viability of some organs currently donated (e.g. kidneys), but would likely yield less organs than Option 3. Here we have assumed 1.7 organs per additional donor.
7. This is based upon an assumption that shorter periods of asystole would allow donation of heart and lungs (at a ratio similar to HB donors) from DCD donors.

## Footnotes

1 SchneiderCEJesting PilateHastings Cent Rep20083814–151870991310.1353/hcr.0.0038; J. McKinley. 2008. Surgeon Accused of Speeding a Death to Get Organs. *New York Times* 27 Feb. Available at: http://www.nytimes.com/2008/02/27/us/27transplant.html[Accessed 28 Jan 2009]; S. Chawkins. 2008. Transplant Surgeon Acquitted in Case Involving Potential Organ Donor's Death. *Los Angeles Times* 19 Dec. Available at: http://www.latimes.com/features/health/la-me-transplant19-2008dec19,0,2830878.story[Accessed 28 Jan 2009] There are a number of ethical concerns that might be raised about organ donation in this particular case. There was a failure of the usual separation between the treating team and the transplant team. It is not clear whether his prognosis warranted withdrawal of life support. His mother was not present when he died, and may not have understood the details of the organ donation that she was agreeing to. These will be discussed briefly in Section F.

2 SmithMMurphyPA Historic Opportunity to Improve Organ Donation Rates in the UkBr J Anaesth2008100735–7371848310910.1093/bja/aen124

3 SteinbrookROrgan Donation after Cardiac DeathN Engl J Med2007357209–2131763445510.1056/NEJMp078066

4 CallaghanCJBradleyJACurrent Status of Renal TransplantationMethods Mol Biol20063331–281679084510.1385/1-59745-049-9:1

5 Smith & Murphy, *op. cit.* note 2; Steinbrook, *op. cit*. note 3.

6 United Network for Organ Sharing (UNOS). 2009. *United Network for Organ Sharing: Organ donation and transplantation*, Richmond, Virginia available at http://www.unos.org[Accessed 07 Jan 2009].

7 NHS Blood and Transplant. Transplant Activity in the UK 2008–9. August 2009. Available at http://www.uktransplant.org.uk/ukt/statistics/transplant_activity_report/transplant_activity_report.jsp[Accessed 25 Nov 09].

8 Smith & Murphy. *op. cit.* note 2; BayatSMedical and Non-medical Determinants of Access to Renal Transplant Waiting List in a French Community-based Network of CareNephrol Dial Transplant2006212900–29071686124510.1093/ndt/gfl329. Of note, the UK figures for deaths on the transplant waiting list are approximately ^1^/_2_ those of the United States (adjusted for population difference). This is not likely to be due to a higher number of transplants in the UK. In 2008–9 there were approximately 59 solid organ transplants per million population in the UK, (NHS Blood and Transplant, *op. cit*. note 7) while in 2007 there were approximately 92 solid organ transplants per million population in the US (2008 OPTN/SRTR Annual Report: transplant data 1998–2007 Available at http://www.ustransplant.org/annual_reports/current/default.htm[Accessed 02 Dec 09].

9 BrookNRWallerJRNicholsonMLNonheart-Beating Kidney Donation: Current Practice and Future DevelopmentsKidney Int2003631516–15291263136910.1046/j.1523-1755.2003.00854.x; CooperDKXenotransplantation – State of the ArtFront Biosci19961248–26510.2741/a1309159232

10 MacdonaldPHeart Transplantation: Who Should Be Considered and When?Intern Med J200838911–9171912054810.1111/j.1445-5994.2008.01833.x

11 AbadieAGaySThe Impact of Presumed Consent Legislation on Cadaveric Organ Donation: A Cross-Country StudyJ Health Econ200625599–6201649026710.1016/j.jhealeco.2006.01.003; QuigleyMThe Organs Crisis and the Spanish Model: Theoretical Versus Pragmatic ConsiderationsJ Med Ethics200834223–2241837566910.1136/jme.2007.023127

12 Abadie & Gay. *op. cit*. note 11; RithaliaAImpact of Presumed Consent for Organ Donation on Donation Rates: A Systematic ReviewBMJ2009338a31621914747910.1136/bmj.a3162PMC2628300

13 VeatchRMDonating Hearts after Cardiac Death – Reversing the IrreversibleN Engl J Med20083596721870346810.1056/NEJMp0805451; WijdicksEFBrain Death Worldwide: Accepted Fact but No Global Consensus in Diagnostic CriteriaNeurology20025820–251178140010.1212/wnl.58.1.20; M. Potts. 2007. A non-sequitur (e-letter) *J Med Ethics.* London, UK. Available at http://jme.bmj.com/content/33/4/197/reply[Accessed 7 Jan 2009].

14 DorlingAClinical Xenotransplantation of Solid OrgansLancet1997349867–871912127510.1016/S0140-6736(96)09404-4

15 For example see ShapiroRSFuture Issues in Transplantation Ethics: Ethical and Legal Controversies in Xenotransplantation, Stem Cell, and Cloning ResearchTransplant Rev (Orlando)2008222102141863188110.1016/j.trre.2008.04.004; SpitalAIncreasing the Pool of Transplantable Kidneys through Unrelated Living Donors and Living Donor Paired ExchangesSemin Dial2005184694731639870810.1111/j.1525-139X.2005.00091.x; PasquerellaLSmithSLaddRInfants, the Dead Donor Rule, and Anencephalic Organ Donation: Should the Rules Be Changed?Med Law20012041742311713840; VeatchRMAbandon the Dead Donor Rule or Change the Definition of Death?Kennedy Inst Ethics J2004142612761549722610.1353/ken.2004.0035; Radcliffe-RichardsJThe Case for Allowing Kidney Sales. International Forum for Transplant EthicsLancet199835119501952965427910.1016/s0140-6736(97)08211-1

16 UK Transplant. 2008. *Potential Donor Audit (Summary Report for the 24 month period 1 April 2006 – 31 March 2008).* Bristol, UK. Available at http://www.uktransplant.org.uk/ukt/statistics/potential_donor_audit/potential_donor_audit.jsp[Accessed 7 Jan 2009]

17 RidleySUK Guidance for Non-Heart-Beating DonationBr J Anaesth2005955925951618368310.1093/bja/aei235

18 The vast majority of patients from whom life support is withdrawn die, though the interval between withdrawal and death varies considerably. In practice a very small proportion of patients survive after treatment is withdrawn, often with significant impairments and an abbreviated lifespan. LewisJPHoKMWebbSAOutcome of patients who have therapy withheld or withdrawn in ICUAnaesth Intensive Care2007353873921759113410.1177/0310057X0703500312; NolinTAnderssonRWithdrawal of Medical Treatment in the ICU. A Cohort Study of 318 Cases During 1994–2000Acta Anaesthiol Scand20034750150710.1034/j.1399-6576.2003.00128.x12699505; EstebanAWithdrawing and Withholding Life Support in the Intensive Care Unit: A Spanish Prospective Multi-Centre Observational StudyIntensive Care Med200127174417491181011710.1007/s00134-001-1111-7; O'CallahanJGWithholding and Withdrawing of Life Support from Patients with Severe Head InjuryCrit Care Med19952315671575766456010.1097/00003246-199509000-00018. For example in one Australian study, 9 (2.3%) of 396 patients who had decisions to limit life support in intensive care survived to hospital discharge. Of these patients only 2 (0.6%) had prolonged survival – both with severe neurological impairments. (Lewis, *op. cit.* this note.) In another Spanish study none of 226 patients who had life support withdrawn survived to hospital discharge. (Esteban, *op. cit.* this note.) We assume in what follows that one criterion for a patient being a LSW donor is a very high probability of death (following treatment withdrawal).

19 ZandbergenESystematic Review of Early Prediction of Poor Outcome in Anoxic Ischaemic ComaLancet199835218081812985138010.1016/S0140-6736(98)04076-8

20 VerheijdeJRadyMMcgregorJRecovery of Transplantable Organs after Cardiac or Circulatory Death: Transforming the Paradigm for the Ethics of Organ DonationPhilos Ethics Humanit Med2007281751903010.1186/1747-5341-2-8PMC1892566; ThomasICabornSManaraAExperiences in the Development of Non-Heart Beating Organ Donation Scheme in a Regional Neurosciences Intensive Care UnitBr J Anaesth20081008208261845664210.1093/bja/aen106. DCD has also taken place in patients who have suffered an unexpected cardiac arrest, and who are unable to be resuscitated. These instances (sometimes referred to as ‘uncontrolled DCD’) represent a minority of DCD. There has been some speculation about the use of external cardiac compressors to convert uncontrolled into controlled DCD. ZeilerKThe Ethics of Non-Heart-Beating Donation: How New Technology Can Change the Ethical LandscapeJ Med Ethics2008345265291859128710.1136/jme.2007.021568. In this paper we refer exclusively to controlled DCD in LSW donors.

21 MaglioccaJExtracorporeal Support for Organ Donation after Cardiac Death Effectively Expands the Donor PoolJ Trauma200558109511021599545410.1097/01.ta.0000169949.82778.df

22 Smith & Murphy, *op. cit*. note 2. In the UK from 2006-8, brain-dead donors gave 3.9 organs/donor on average, while DCD donors gave 2.7 organs/donor. (UK Transplant Potential Donor Audit, Personal Communication)

23 Steinbrook, *op. cit.* note 3; BernatJLThe Boundaries of Organ Donation after Circulatory DeathN Engl J Med20083596691870346710.1056/NEJMp0804161. It has been estimated that in 20% of cases, organ donation is cancelled because death does not follow quickly enough after withdrawal of life support. (Steinbrook, *op. cit.* note 3.) This may be an underestimate of the total number of patients who die following withdrawal of LST who are ineligible for organ donation because of the length of time it takes for them to die. There is usually an attempt made before approaching families to assess whether it is likely that the patient will die within a given timeframe and some prediction tools have been developed for this purpose. (Bernat, *op. cit*. this note.) In another study of patients having LST withdrawn in intensive care who were potentially eligible for DCD, only 20% died within 1 hour of treatment withdrawal. RevellyJPAre Terminally Ill Patients Dying in the ICU Suitable for Non-Heart Beating Organ Donation?Intensive Care Med2006327087121653456910.1007/s00134-006-0116-7

24 KooglerTCostarinoATJrThe Potential Benefits of the Pediatric Nonheartbeating Organ DonorPediatrics19981011049960623410.1542/peds.101.6.1049

25 RadyMYVerheijdeJLMcGregorJ‘Non-Heart-Beating,’ Or ‘Cardiac Death,’ Organ Donation: Why We Should CareJ Hosp Med200723243341793524310.1002/jhm.204

26 BoucekMMPediatric Heart Transplantation after Declaration of Cardiocirculatory DeathN Engl J Med20083597091870347310.1056/NEJMoa0800660; Magliocca et al., *op. cit.* note 21.

27 Boucek et al., *op. cit.* note 26.

28 Magliocca et al., *op. cit*. note 21; KoWJExtracorporeal Membrane Oxygenation Support of Donor Abdominal Organs in Non-Heart-Beating DonorsClin Transplant2000141521561077042110.1034/j.1399-0012.2000.140209.x

29 Zeiler et al., *op. cit.* note 20

30 R. Herdman & J.T. Potts. 1997. *Non-Heart-Beating Organ Transplantation : Medical and Ethical Issues in Procurement*. Washington, DC: National Academy Press: 57; BernatJLReport of a National Conference on Donation after Cardiac DeathAm J Transplant200662812911642631210.1111/j.1600-6143.2005.01194.x

31 Veatch, *op. cit*. note 13.

32 Bernat, *op. cit.* note 23; Koogler & Costarino Jr., *op. cit.* note 24.

33 Bernat, *op. cit.* note 23.

34 Magliocca et al., *op. cit.* note 21; DejohnCZwischenbergerJEthical Implications of Extracorporeal Interval Support for Organ Retrieval (Eisor)ASAIO Journal2006521191221655709510.1097/01.mat.0000206486.80829.58

35 Magliocca et al., *op. cit.* note 21; Ko et al., *op. cit.* note 28.

36 Bernat. *op. cit.* note 23.

37 The order of principles here is not meant to convey any lexical priority of different principles.

38 One heart, two lungs, two kidneys, split liver (one adult, one child recipient), one pancreas and one intestine.

39 TruogRRobinsonWRole of Brain Death and the Dead-Donor Rule in the Ethics of Organ TransplantationCrit Care Med200331239123961450197210.1097/01.CCM.0000090869.19410.3C

40 Ibid. Truog refers to this principle as ‘respect for persons’.

41 For organ donation the concept of patient autonomy is somewhat different from autonomy in other contexts since the patient is (often) deceased, and the question is whether their previous wishes should be respected.

42 RobertsonJAThe Dead Donor RuleHastings Cent Rep19992961410641238; JoffeAThe Ethics of Donation and Transplantation: Are Definitions of Death Being Distorted for Organ Transplantation?Philos Ethics Humanit Med20072281803625410.1186/1747-5341-2-28PMC2211498

43 Robertson, *op. cit.* note 42.

44 So for example, the British Transplant Society guidelines for DCD stipulates that sufficient time has elapsed after cardiac arrest for hypoxic injury to the brain and brain-stem to have occurred. British Transplant Society. 2005. *Guidelines Relating to Solid Organ Transplants from Non-Heart Beating Donors*. London: BTS. After all brain function has been irreversibly lost we can be confident that they have no further need for their organs. By contrast, the influential US Institute of Medicine paper on DCD argued that it was irreversibility and not brain damage that was relevant to the definition of death on cardiopulmonary criteria. Herdman & Potts, *op. cit.* note 30, p59. We acknowledge that there is substantial overlap between Principles 5 and 6.

45 J.K. Mason & G. T. Laurie. 2005. *Mason and McCall Smith's law and medical ethics.* Oxford: Oxford University Press: 634–635. There are, however good reasons to think that withdrawal of life-sustaining treatment should be classified as an act of killing. F.G. Miller, R.D. Truog & D.W. Brock. Moral Fictions and Medical Ethics. *Bioethics* 2009; published online 7 Jul 2009. DOI: 10.1111/j.1467-8519.2009.01738.x

46 UK Transplant, *op. cit*. note 16.

47 UK Potential Donor audit, ibid.

48 SpitalAErinCAConscription of Cadaveric Organs for Transplantation: Let's at Least Talk About ItAm J Kidney Dis2002396116151187758210.1053/ajkd.2002.32164; SilverTThe Case for a Post-Mortem Organ Draft and a Proposed Model Organ Draft ActBoston Univ Law Rev19896868172811650249

49 Spital & Erin, *op. cit.* note 48.

50 HerringAAWoolhandlerSHimmelsteinDUInsurance Status of US Organ Donors and Transplant Recipients: The Uninsured Give but Rarely ReceiveInt J Health Serv2008386416521906928510.2190/HS.38.4.d

51 It is arguable whether dead people can be used as a mere means in the same way as we use live people as a mere means. They are dead, have no ongoing interests and are incapable of consenting.

52 PierscionekBWhat Is Presumed When We Presume Consent?BMC Med Ethics2008981843924210.1186/1472-6939-9-8PMC2375872

53 Quigley et al., *op. cit.* note 11.

54 The UK organ donation taskforce rejected evidence of possible increases in donor numbers and recently released a report advising against the adoption of opt-out consent in the UK. Organ Donation Taskforce. 2007. *The potential impact of an opt out system for organ donation in the UK: an independent report from the Organ Donation Taskforce.* London, UK. Available at: http://www.dh.gov.uk/en/Publicationsandstatistics/Publications/PublicationsPolicyAndGuidance/DH_090312[Accessed 07 Jan 2009].

55 We are grateful to an anonymous reviewer for encouraging us to investigate this alternative. Combinations of these measures are possible. For example an opt-out organ donation consent system could be introduced without permitting family members to veto donation. This is sometimes referred to as a ‘hard opt-out’ form of organ donation (ibid:12). It is not possible from UK figures to estimate what effect this would have on overall organ supply.

56 Quigley et al., *op. cit.* note 11.

57 UK Transplant audit, *op. cit.* note 16.

58 This depends whether the organs made available were of the right sort and tissue type to meet the needs of those currently dying while waiting for a transplant. It may not be enough to provide organs for those patients who at present are arbitrarily excluded from waiting lists (see note 8).

59 Truog & Robinson, *op. cit.* note 39; C. Coons & N. Levin. The Dead Donor Rule, Voluntary Active Euthanasia and Capital Punishment. *Bioethics* 2009. Published online 26 Oct 2009. DOI: 10.1111/j.1467-8519.2009.01767.x

60 Veatch, *op. cit.* note 15.

61 P. Singer. 1994. *Rethinking Life and Death*. Melbourne: The Text Publishing Company: 207. In two surveys in the US, a majority of respondents supported donation of organs from patients in permanent coma or vegetative state. DuBoisJMSchmidtTDoes the Public Support Organ Donation Using Higher Brain-Death Criteria?J Clin Ethics200314263612953351; SiminoffLABurantCYoungnerSJDeath and Organ Procurement: Public Beliefs and AttitudesSoc Sci Med200459232523341545070710.1016/j.socscimed.2004.03.029

62 J. Rachels. 1986. *The End of Life : Euthanasia and Morality*. Oxford: Oxford University Press: 24–25.

63 Truog & Robinson, *op. cit.* note 39.

64 Ibid. See also Section F.

65 See for example ibid; ArnoldRMYoungnerSJThe Dead Donor Rule: Should We Stretch It, Bend It, or Abandon It?Kennedy Inst Ethics J199332632781012653810.1353/ken.0.0153. FostNReview: The New Body Snatchers: On Scott's ‘The Body as Property’Am Bar Found Res J19838718732

66 Truog's justification for ODE is different from that presented here. He argues that current concepts of brain death and the dead-donor rule are incoherent, and he proposes an alternative based upon the principles of autonomy and non-maleficence. We find Truog's arguments compelling. Our paper can be seen as providing a complementary argument in favour of ODE. Truog favours a narrow definition for the group of patients who may consent to this procedure: only those who will die within minutes of withdrawal of life support, or who are permanently unconscious. Our definition of LSW donors overlaps with Truog's, but includes the larger group of patients from whom it is permissible to withdraw life support in intensive care, and whose death is highly likely to ensue (though not necessarily instantly).

67 A. Buchanan. 1985. *Ethics, Efficiency and the Market*. Oxford: Clarendon Press: 12.

68 Ibid; ShawPThe Pareto Argument and InequalityPhilos Q199949353368

69 Ibid.

70 WilkinsonDJFunctional Neuroimaging and Withdrawal of Life-Sustaining Treatment from Vegetative PatientsJ Med Ethics2009355085111964401010.1136/jme.2008.029165PMC2711351

71 DetryOOrgan Donation after Physician-Assisted DeathTranspl Int2008219151853792410.1111/j.1432-2277.2008.00701.x

72 In the Belgian case 10 minutes elapsed after the circulation ceased before organ retrieval commenced. Only the liver and kidneys were transplanted. (Ibid.)

73 Wijdicks *op. cit.* note 13.

74 Magliocca et al., *op. cit.* note 21.

75 Absent kidney, liver or pancreatic function can lead to death – but not usually in a short time frame (days or weeks rather than hours). Thus if death were predicted within this time frame, organ donation prior to treatment withdrawal would be unlikely to affect the timing of death.

76 It may be possible to reduce the chance of this – for example with the use of epidural anaesthesia.

77 Magliocca et al., *op. cit.* note 21.

78 Dejohn & Zwischenberger, *op. cit.* note 34.

79 DeVitaMASnyderJVDevelopment of the University of Pittsburgh Medical Center Policy for the Care of Terminally Ill Patients Who May Become Organ Donors after Death Following the Removal of Life SupportKennedy Inst Ethics J199331311431012652610.1353/ken.0.0175

80 Boucek et al., *op. cit.* note 26.

81 If the interval were shortened to less than one minute it would not be possible to distinguish extreme bradycardia from periods of asystole.

82 ShemieSClarifying the Paradigm for the Ethics of Donation and Transplantation: Was ‘Dead’ Really So Clear before Organ Donation?Philos Ethics Humanit Med20072181771891810.1186/1747-5341-2-18PMC2048971

83 OzarkSDevitaMANon-Heartbeating Organ Donation: Ethical Controversies and Medical ConsiderationsInt Anesthesiol Clin2001391031161152460310.1097/00004311-200107000-00009

84 Although organ donation after brief asystole might lead to death *sooner* than would otherwise occur, it would not be likely to lead to death in patients who *would have survived* had organ donation not taken place. (see note 18) Even if the heart restarts in this setting, without further resuscitation the death of the patient is likely to follow within a fairly short interval.

85 Except option 1c – increased efficiency of obtaining consent.

86 Verheijde, Rady & Mcgregor, *op. cit.* note 20.

87 Indeed, some may have concerns that even the discussion of such options may negatively impact on organ donation rates.

88 In a similar vein some some have supported the idea that individuals should be permitted to opt-in to alternative criteria for death. ZeilerKDeadly pluralism? Why death concept, death definition, death criterion and death test pluralism should be allowed, even though it creates some problemsBioethics2009234504591855427710.1111/j.1467-8519.2008.00669.x. BagheriAIndividual Choice in the Definition of DeathJ Med Ethics2007331461491732938410.1136/jme.2006.016014PMC2598255. Veatch. Abandon the Dead Donor Rule or Change the Definition of Death? op. cit. note 15.

89 Indeed, many current donors might not be aware of all available means of organ donation (for example DCD). Our proposal would increase autonomy by providing patients with a range of options and allowing them to choose those they wished to apply to themselves.

90 In the case of Ruben Navarro there was a break-down in this normal separation.

91 A requirement for independent confirmation of prognosis for LSW donors would potentially delay treatment withdrawal. But at present treatment withdrawal is sometimes delayed to allow transplant teams to prepare. There would be a strong incentive to ensure that confirmation of prognosis was able to occur expeditiously. If this were not able to take place within a reasonable time frame life support might still be able to be withdrawn, but organ donation could not proceed.

92 J. Bourke. 2006. *Fear : A Cultural History*. London: Virago: 317.

93 As noted earlier (note 61), there is a relatively high level of community support for the latter, though it has not been adopted in any jurisdiction. We are grateful to an anonymous reviewer for highlighting this objection.

94 Organ Donation Euthanasia would potentially be a Pareto improvement in jurisdictions where voluntary active euthanasia is permitted and a patient has consented to euthanasia (for reasons other than the donation of their organs), Coons & Levin, *op. cit.* note 59. However, again, this does not in itself settle the question of whether voluntary active euthanasia (for patients who are not dependent on life support) should be permitted (ibid.).

95 See note 1.

96 UK Transplant, *op. cit.* note 16.

97 NHS Blood and Transplant, *op. cit*. note 7.

98 UK Potential Donor Audit, *op. cit.* note 22.

99 Smith & Murphy, *op. cit*. note 2.

100 UK Potential Donor Audit 1 April 2008-31 March 2009 (personal communication 27 Nov 2009).

101 See note 23.

102 Revelly, *op. cit.* note 23.

